# Function drives 1‐year decision regret in radical prostatectomy: a multicentre prospective study

**DOI:** 10.1111/bju.70382

**Published:** 2026-07-10

**Authors:** Christopher Hirtsiefer, Emma Kümmel, Anna Vogelgesang, Fabian Falkenbach, Mona Kafka, Annemarie Uhlig, Tim Nestler, Cem Aksoy, Iva Simunovic, Johannes Huber, Isabel Heidegger, Markus Graefen, Marianne Leitsmann, Christian Thomas, Martin Baunacke

**Affiliations:** ^1^ Department of Urology University Hospital Dresden Dresden Germany; ^2^ Martini‐Klinik Prostate Cancer Centre University Hospital Hamburg‐Eppendorf Hamburg Germany; ^3^ Department of Urology Medical University Innsbruck Innsbruck Austria; ^4^ Department of Urology University Medical Center Göttingen Göttingen Germany; ^5^ aQua – Institute for Applied Quality Improvement and Research in Health Care Göttingen Germany; ^6^ Department of Urology Federal Armed Forces Hospital Koblenz Koblenz Germany; ^7^ Department of Urology Phillips‐University Marburg Marburg Germany; ^8^ Department of Urology Medical University of Graz Graz Austria; ^9^ Department of Urology University Hospital Heidelberg Heidelberg Germany; ^10^ Vancouver Prostate Centre, M.H. Mohseni Institute of Urologic Sciences University of British Columbia Vancouver British Columbia Canada

**Keywords:** decision regret, radical prostatectomy, functional outcomes, postoperative erectile function, surgical approach, symptom severity, postoperative functional satisfaction

## Abstract

**Objectives:**

To identify contemporary drivers of postoperative decision regret 1 year after radical prostatectomy (RP).

**Patients and Methods:**

In this prospective, multicentre study, patients undergoing robot‐assisted RP (RARP) or retropubic open RP (ORP) for localised prostate cancer at seven centres in Germany and Austria (2022–2024) completed questionnaires preoperatively and at 1 year postoperatively. Decision regret was assessed using the Decision Regret Scale (DRS). Functional outcomes were measured with European Organisation for Research and Treatment of Cancer quality of life questionnaire – 25‐item prostate cancer specific (EORTC QLQ‐PR25) symptom scores at 1 year postoperatively. Multivariable logistic regression identified predictors of high surgical decision regret (DRS score ≥10). Sensitivity was tested via propensity score matching (PSM).

**Results:**

A total of 419 patients completed the surveys (58% RARP, 42% ORP) at 1 year postoperatively. Surgical decision regret was low (mean [SD] DRS score 9.0 [15.4]). Erectile dysfunction was the outcome that most patients self‐reported as worse than expected (54% [221/404]), followed by urinary incontinence (23% [96/414]) and oncological cure (10% [39/397]) in a questionnaire on their retrospective perception. The ORP patients reported higher regret regarding surgical approach than RARP patients (mean [SD] DRS score 11.3 [17.1] vs 7.4 [13.9], *P* = 0.02). In a multivariate analysis, high urinary symptom burden (odds ratio [OR] 2.4, *P* = 0.009) and impaired sexual functioning (OR 2.8, *P* = 0.002) independently predicted high surgical decision regret whereas surgical approach and prostate‐specific antigen recurrence did not. Sensitivity analyses via PSM confirmed these independent predictors.

**Conclusions:**

Surgical decision regret after RP is primarily driven by postoperative functional outcomes rather than oncological results or surgical approach. Optimising functional recovery and managing patient expectations are key strategies to reduce regret in prostate cancer surgery.

**Abbreviations**
DRS: Decision Regret Scale
EORTC QLQ‐PR25: European Organisation for Research and Treatment of Cancer quality of life questionnaire – 25‐item prostate cancer specific
OR: odds ratio
PCa: prostate cancer
PSM: propensity score matching
QoL: quality of life
(O)(RA)RP: (open) (robot‐assisted) radical prostatectomy
UICC: Union Internationale Contre le Cancer

## Introduction

Patients with localised prostate cancer (PCa) are presented with a variety of treatment choices ranging from active surveillance to radiotherapy and radical prostatectomy (RP) and focal treatment in clinical trials. The techniques of RP have seen an overwhelming shift in modern healthcare systems with robot‐assisted RP (RARP) being the most frequently performed robotic procedure [1]. Nonetheless, depending on the healthcare system, both surgical procedures may be offered equally, or open RP (ORP) may be the dominant procedure with advantages in accessibility, procedure length and cost of index hospitalisation [2, 3, 4].

Even though oncological outcome has been shown to be similar for both techniques [4, 5], a previous study of our group demonstrated that RP patients are not well informed about this oncological equivalence of RARP and ORP [6]. This was striking as they referred to urologists and surgical centres as their most important sources of information. We investigated risk factors for this misconception finding that older, less educated RARP patients are at higher risk of wrongfully conceiving their procedure as superior [6].

The finding of wrongful preoperative beliefs raised the question of how they view it in retrospect and its impact on postoperative satisfaction with their decision. This metric has previously been studied as ‘decision regret’ [7, 8, 9, 10] measured by the Decision Regret Scale (DRS) [11]. The DRS is a validated patient‐reported outcome measure that quantifies distress or remorse after a healthcare decision, with higher scores indicating greater treatment‐related regret. Functional and quality‐of‐life (QoL) measures have repeatedly been shown to supersede PSA recurrence in their impact on the DRS [7, 10, 12]. At a time of somewhat balanced robotic and open procedures in German urology departments by 2018 [1], decision regret of RP patients was shown to depend most on erectile and voiding function and the surgical technique had no influence on postoperative regret [10]. In the 7 years since, however, the predominance of RARP as well as the general adaption and public acceptance of robotic surgery has increased, with robot‐assisted surgery now being perceived as more precise than open surgery [13]. The number of studies investigating decision regret, also regarding surgical technique, decreased, with newer studies either ignoring subdifferentiation or focusing purely on RARP and often lacking preoperative data beyond tumour characteristics [14, 15, 16].

Given the findings of misconceptions preoperatively and the paucity of current comparative data including both techniques, this study followed up on a multicentric cohort initially bearing misconceptions and reveals up‐to‐date developments in PCa decision regret, investigating patient and procedure characteristics, as well as decisional and behavioural factors.

## Patients and Methods

This prospective study was conducted across seven urology departments in Germany and Austria between 2022 and 2024. Four centres offered both ORP and RARP, two exclusively ORP and one exclusively RARP (Table S6).

Patients with clinically localised or node‐positive primary PCa (Union Internationale Contre le Cancer [UICC] stage <IVB) and scheduled for either RARP or ORP were included. Patients unable to consent and having metastasised (UICC stage IV) or recurrent disease (salvage) were excluded. Participants completed the first questionnaire prior to their pre‐surgical consultation (Appendix S1). Dispatching of the self‐reported surveys via mail 1 year after surgery was independent of follow‐ups by centre and practitioners mandated by German guidelines. Reminders were sent upon 6 weeks of a missing reply. The methodology of this framework project has previously been published with our preoperative studies [6]. This study focuses on the 1‐year postoperative questionnaires (Appendix S2).

Preoperative data included patient‐reported expectations of outcomes as well as patient, tumour, and surgery characteristics recorded by the centre. Information on nerve sparing was not recorded.

Postoperative data includes patient‐reported decision regret concerning the choice of therapy (external beam radiation therapy ± hormonal therapy vs RP vs active surveillance, if applicable) and decision regret concerning the surgical technique (RARP vs ORP), as well as QoL metrics.

The DRS [11] is a validated patient‐reported outcome measure that quantifies distress or remorse after a healthcare decision, testing five categories of regret with higher scores resembling higher regret. The final score ranges 0–100. In the absence of pre‐defined cut‐off values for the DRS, we created two groups for high (≥10) and low (<10) decision regret along the median in our cohort in line with previous studies [8, 17]. The QoL metrics, quantified by the European Organisation for Research and Treatment of Cancer quality of life questionnaire – 25‐item prostate cancer specific (EORTC QLQ‐PR25) Symptom Scores [18], for urinary, bowel and hormonal symptoms, as well as sexual activity, sexual functioning and the use of incontinence aids, were recorded preoperatively and again at 1 year postoperatively. Preoperative expectations of outcomes were followed up by asking for retrospective assessments of the chosen therapy's performance in oncological control, functional results, complications, wound healing, catheter duration, hospitalisation, and convalescence. Patients were asked to evaluate their procedure as having performed better, worse or exactly as preoperatively expected. Similarly, patients were asked on their retrospective perception of their decisional and informative behaviour preoperatively.

Data were analysed via the chi‐squared test and *t*‐test. Logistic regression models were used for multivariable analyses. The parameters with statistical significance in univariate analyses were included in the multivariate analysis. A *P* < 0.05 was considered to indicate statistical significance.

Additionally, a propensity score matched (PSM) sensitivity analysis was performed to compare findings to cohorts with reduced baseline imbalances between ORP and RARP patients. Nearest‐neighbour matching with a 1:1 ratio, a calliper width of 0.1, and matching with replacement was applied using age, D'Amico risk group, and monthly household income as matching variables. For PSM, only centres offering both surgical approaches were included to ensure sufficient overlap and enable valid matching between treatment groups. Because matching with replacement was used, subsequent analyses were performed using propensity score weights within a survey‐weighted framework. Group forming of the main analyses was repeated in the matched cohort.

Missing data were excluded per variable for tests. For logistic regression models and sensitivity analyses, missing cases in any model variable led to exclusion from the model. Valid case numbers are reported among the tables. All statistical analyses were performed using RStudio (version 2026.04.0 + 526, Posit Software, PBC, Boston, MA, USA) for R (R version 4.4.1, R Foundation for Statistical Computing, Vienna, Austria).

Ethics committee approval was obtained in advance by the Ethic Committee of the Technical University of Dresden (BO‐EK‐438092021). All study participants have provided informed consent.

## Results

### Cohort

Initially 508 patients were recruited in seven centres. In all, 419 patients (ORP: 42% [174/419], RARP: 58% [245/419]) answered both questionnaires (response rate: 82% [419/508]). The majority of patients were classified as D'Amico intermediate risk (60% [250/419]), but more ORP patients had high‐risk tumours (35% [60/174] vs 14% [34/245], *P* < 0.01). Both groups were of similar mean age (ORP: 65.1 years, RARP: 64.6 years, *P* = 0.4) and education (*P* = 0.053). RARP patients more often had private health insurance (33% [79/245] vs 22% [36/174], *P* = 0.02), consistent with their generally higher household income (>4000 €/month: 40% [87/245] vs 29% [46/174], *P* = 0.03) (Table 1).

**Table 1:** Study sample by surgical approach with outcome and quality of life (*n* = 419).

Variable	All (*n* = 419)	ORP (*n* = 174)	RARP (*n* = 245)	*P*
Age at surgery, years, mean (SD); median [range]	64.8 (6.7); 65 [45–78]	65.1 (6.7); 65 [47–78]	64.6 (6.7); 65 [45–78]	0.4
D'Amico risk group (*n* = 414), *n* (%)				
Low	70 (18)	23 (14)	47 (19)	**<0.001**
Intermediate	250 (60)	88 (51)	162 (67)	
High	94 (22)	60 (35)	34 (14)	
Preoperative PSA level, ng/mL, mean (SD); median [range]	9.5 (9.9); 6.5 [0.1–94.9]	10.4 (10.0) 6.8 [0.1–62.8]	8.9 (9.9); 6.4 [0.1–94.9]	**<0.01**
Clinical tumour stage (*n* = 414), *n* (%)				
T1	294 (71)	109 (68)	185 (73)	0.428
T2	103 (25)	44 (27)	59 (23)	
T3	16 (4)	7 (4)	9 (4)	
T4	1 (0)	1 (1)	0 (0)	
Clinical lymph node stage (*n* = 412), *n* (%)				
N0	341 (83)	140 (87)	201 (80)	**<0.01**
N1	14 (3)	9 (6)	5 (2)	
Nx	57 (14)	12 (7)	45 (18)	
Insurance (*n* = 400), *n* (%)				
Statutory	285 (71)	127 (78)	158 (67)	**0.02**
Private	115 (29)	36 (22)	79 (33)	
Residential area (*n* = 409), *n* (%)				
Rural	197 (48)	86 (51)	111 (46)	0.4
Urban	212 (52)	84 (49)	128 (54)	
Marital status (*n* = 410), *n* (%)				
Single	38 (9)	17 (10)	21 (9)	0.7
Married	372 (91)	154 (90)	218 (91)	
Education (*n* = 366), *n* (%)				
None	1 (0.3)	1 (1)	0 (0)	0.053
High school (Hauptschule)	95 (26)	45 (30)	50 (23)	
High school (Realschule)	123 (34)	54 (36)	69 (32)	
Baccalaureate	147 (40)	48 (33)	99 (45)	
Monthly household income (EUR) (*n* = 376), *n* (%)				
<1500 €	27 (7)	16 (10)	11 (5)	**0.03**
1500–4000 €	216 (58)	97 (61)	119 (55)	
>4000 €	133 (35)	46 (29)	87 (40)	
Centre distribution, *n* (%)				
1	132 (31)	46 (26)	86 (35)	*
2	43 (10)	0 (0)	43 (18)	
3	43 (10)	43 (25)	0 (0)	
4	80 (19)	21 (12)	59 (24)	
5	44 (11)	13 (8)	31 (13)	
6	44 (11)	44 (25)	0 (0)	
7	33 (8)	7 (4)	26 (10)	
PSA recurrence ≥0.2 ng/mL (*n* = 394), *n* (%)				
Yes	26 (7)	16 (10)	10 (4)	**0.03**
No	368 (93)	145 (90)	223 (96)	
Urinary incontinence (*n* = 193), *n* (%)				
Yes	26 (7)	16 (10)	10 (4)	0.3
No	368 (93)	145 (90)	223 (96)	
EORTC score, mean (SD); median [range]				
Urinary symptoms (*n* = 375)	28.8 (18.9); 25.0 [0–83.3]	29.5 (19.4); 25.0 [0–79.2]	28.3 (18.7); 25.0 [0–83.3]	0.6
Bowel symptoms (*n* = 378)	7.5 (12.6) 0 [0–83.3]	8.5 (14.4) 0 [0–83.3]	6.8 (11.2) 0 [0–66.7]	0.2
Hormone symptoms (*n* = 325)	15.4 (13.6) 11.1 [0–72.2]	15.5 (13.1) 11.1 [0–61.1]	15.3 (14.0) 11.1 [0–72.2]	0.9
Sexual activity (*n* = 405)	46.0 (28.4) 50.0 [0–100]	41.5 (28.7) 33.3 [0–100]	49.1 (27.9) 50.0 [0–100]	**0.008**
Sexual functioning symptoms (*n* = 220)	47.8 (22.9) 50.0 [0–100]	48.8 (22.0) 50.0 [8.3–100]	47.3 (23.4) 41.7 [0–100]	0.6

* Statistical testing not applicable.

Bold values statistically significant at *P* < 0.05.

Biochemical recurrence (PSA rise of >0.2 ng/mL) occurred in 7% (26/394) of patients after 1 year (median follow‐up: 372 days; ORP: 374 days, RARP: 371 days, *P* = 0.57), with more ORP patients being affected (10% [16/174] vs 4% [10/245], *P* = 0.03). Functional outcomes concerning continence were comparable both for incontinence aids (two or more pads: ORP: 20% [16/82] vs 14% [16/111], *P* = 0.3; EORTC incontinence aids: *P* = 0.2) and the mean EORTC urinary symptom score (mean [SD] 29.5 [19.4] vs 28.3 [18.7], *P* = 0.6). Similarly, the EORTC scores showed no significant differences in bowel (*P* = 0.2) or hormone (*P* = 0.9) symptoms for both procedures. Even though there was no advantage for either procedure in the domain of sexual functioning (mean [SD] score 48.8 [22.0] vs 47.3 [23.4], *P* = 0.6), RARP patients did show a higher rating of sexual activity postoperatively (mean [SD] score 49.1 [27.9] vs 41.5 [28.7], *P* < 0.008) (Table 1).

### Decision Regret and Retrospective Assessment by Surgical Approach

**Fig. 1:**
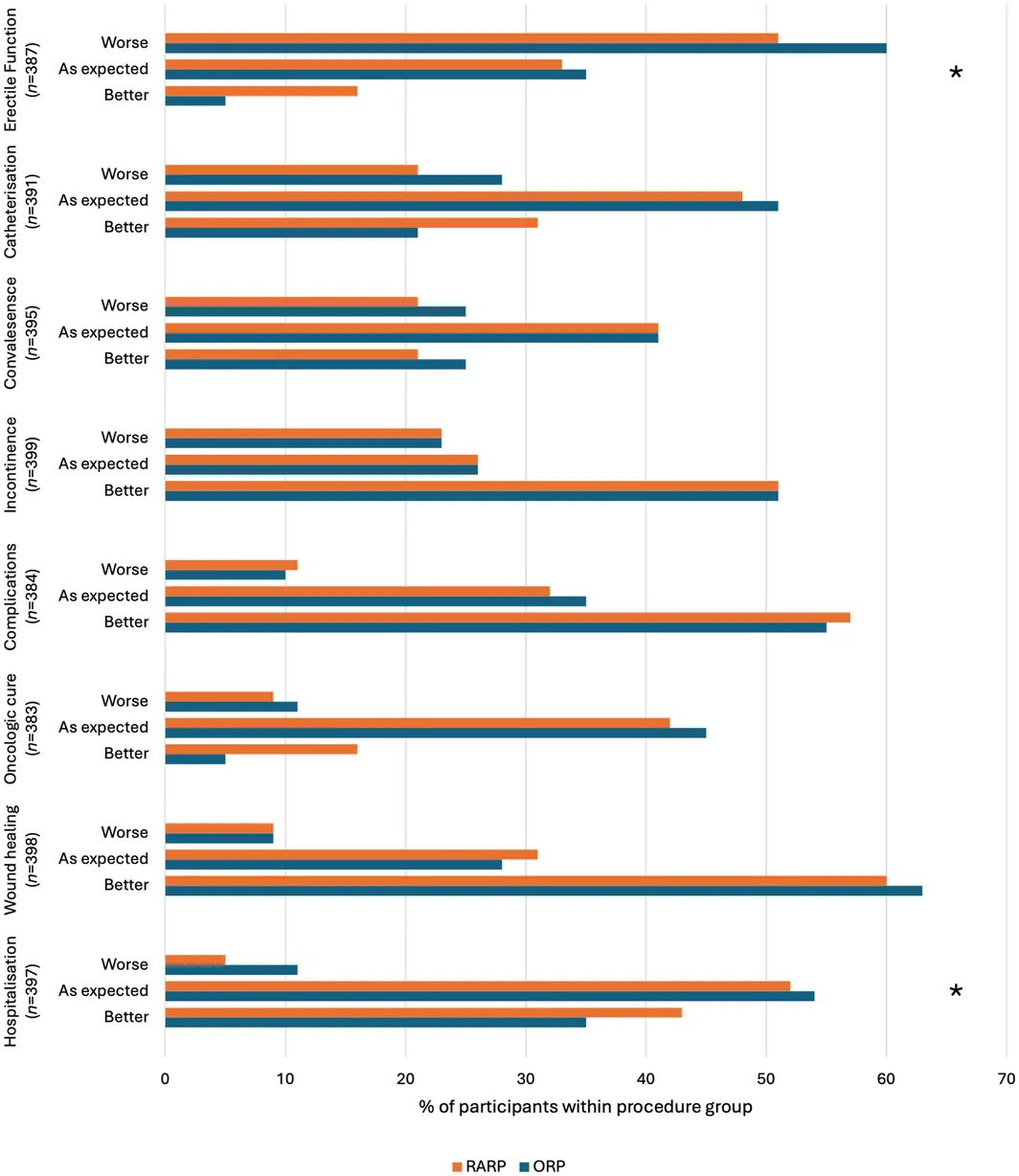
Patients’ postoperative evaluations of outcomes in relation to preoperative expectations grouped by surgical procedure. *P < 0.05, chi‐squared test.

Patients who underwent ORP showed a higher DRS score for the choice of the surgical procedure than RARP patients (mean [SD] score 11.3 [17.1] vs 7.4 [13.9], *P* = 0.02), but not for the general choice of a surgical approach (mean [SD] score 9.5 [15.1] vs 10.4 [16.6], *P* = 0.6). The procedural choice had no influence on the postoperative satisfaction of patients’ preoperative expectations of curation (*P* = 0.6) or urinary continence (*P* = 1.0). However, for more ORP than RARP patients, postoperative erectile function was worse than preoperatively expected (60% [100/168] vs 51% [121/236], *P* = 0.004), with those enjoying better function than expected amounting to 16% in RARP and only 5% in ORP patients (Fig. 1). Similarly, length of index hospitalisation was better or as expected for 95% (231/242) of RARP patients, but only 89% (152/171) for ORP patients (*P* = 0.02). Erectile dysfunction was the outcome that most patients rated as worse than expected (54% [221/404]) with more ORP patients having this opinion (60% [100] vs 51% [121], *P* < 0.01). This retrospective assessment of postoperative erectile function varied by age group with more patients aged >65 years assessing it as worse than expected (113 [57%] vs 108 [52%]; *P* = 0.046). In all, 23% (96/414) of patients rated urinary incontinence as worse than expected and only 10% (39/397) rated oncological outcome as worse than expected, with no approach‐wise differences for either (*P* = 1.0 and *P* = 0.6, respectively; Table S1).

### Surgical Decision Regret

To explore the causes of high decision regret, two groups were formed within the cohort defined by low (DRS score <10, *N* = 277) and high (DRS score ≥10, *N* = 124) surgical decision regret. Within the group with low decision regret, more patients underwent RARP (low decision regret: 70% [194/277] vs high decision regret: 50% [32/124], *P* = 0.02). Risk grouping did not differ between decision regret groups (*P* = 0.2). Patients with low decision regret less frequently used two or more incontinence pads (10% [12/120] vs 27% [16/55], *P* < 0.001) and had a lesser strain of urinary (mean [SD] score 25.6 [18.2] vs 36.0 [18.5], *P* < 0.001) and hormonal (mean [SD] score 13.6 [13.4] vs 19.4 [13.6], *P* < 0.001) symptoms. Low decision regret patients had lower sexual functioning symptoms (mean [SD] score 43.3 [22.1] vs 57.9 [22.1], *P* < 0.001) and a higher sexual activity than high decision regret patients (mean [SD] score 48.7 [28.5] vs 42.0 [28.2], *P* = 0.03). PSA recurrence rates did not differ for low or high surgical decision regret (6% [7/114] vs 7% [18/264], *P* = 0.8; Table 2).

**Table 2:** Study sample by surgical decision regret (DRS score) grouping with outcome and quality of life (*n* = 410).

Variable	All (*n* = 401)	Low surgical decision regret (DRS score <10) (*n* = 277)	High surgical decision regret (DRS score ≥10) (*n* = 124)	*P*
Age at surgery, years, mean (SD); median (range)	64.7 (6.7) 65 [45–78]	64.4 (6.7) 65 [45–78]	65.4 (6.6) 67 [50–78]	0.2
D'Amico risk group (*n* = 397), *n* (%)				
Low	66 (17)	50 (18)	16 (13)	0.5
Intermediate	243 (61)	167 (61)	76 (63)	
High	88 (22)	59 (21)	29 (24)	
Preoperative PSA level, ng/mL, mean (SD); median [range]	9.4 (9.8) 6.7 [0.1–94.9]	9.4 (9.6) 6.6 [0.1–94.9]	9.6 (10.4) 6.7 [0.2–89.0]	0.8
Clinical tumour stage (*n* = 395), *n* (%)				
T1	281 (71)	201 (74)	80 (65)	0.3
T2	98 (25)	61 (22)	37 (30)	
T3	16 (4)	10 (4)	6 (5)	
Clinical lymph node stage (*n* = 394), *n* (%)				
N0	324 (82)	220 (81)	104 (85)	0.3
N1	14 (4)	8 (3)	6 (5)	
Nx	56 (14)	43 (16)	13 (11)	
Insurance (*n* = 383), *n* (%)				
Statutory	273 (71)	188 (70)	85 (73)	0.6
Private	110 (29)	79 (30)	31 (27)	
Residential area (*n* = 391), *n* (%)				
Rural	188 (48)	131 (48)	57 (48)	0.9
Urban	203 (52)	140 (52)	63 (52)	
Marital status (*n* = 392), *n* (%)				
Single	36 (9)	21 (8)	15 (12)	0.6
Married	356 (91)	251 (92)	105 (88)	
Education (*n* = 351), *n* (%)				
None	1 (0.3)	1 (0.4)	0 (0)	0.3
High school (Hauptschule)	93 (26)	58 (24)	35 (33)	
High school (Realschule)	118 (34)	83 (34)	35 (33)	
Baccalaureate	139 (40)	103 (42)	36 (34)	
Monthly household income (EUR) (*n* = 359), *n* (%)				
<1500 €	26 (7)	16 (6)	10 (10)	0.5
1500–4000 €	204 (57)	145 (57)	59 (57)	
>4000 €	129 (36)	95 (37)	34 (33)	
Second opinion used (*n* = 385), *n* (%)				
Yes	198 (51)	135 (51)	63 (53)	0.7
No	187 (49)	131 (49)	56 (47)	
Decisional behaviour (*n* = 381), *n* (%)				
Autonomous	122 (32)	83 (31)	39 (34)	0.9
Shared	237 (62)	166 (63)	71 (61)	
Paternalistic	22 (6)	16 (6)	6 (5)	
Surgical method (*n* = 401), *n* (%)				
ORP	165 (41)	103 (37)	62 (50)	**0.02**
RARP	236 (59)	174 (63)	62 (50)	
Surgical methods offered (*n* = 401), *n* (%)				
One	122 (30)	83 (30)	39 (31)	0.8
Both	279 (70)	194 (70)	85 (69)	
Preoperative problems holding an erection (EORTC Q23), *n* (%)				
No/little	154 (57)	100 (56)	49 (60)	0.6
Moderate/much	114 (43)	79 (44)	32 (40)	
PSA recurrence ≥0.2 ng/mL (*n* = 378), *n* (%)				
Yes	25 (7)	18 (7)	7 (6)	0.8
No	353 (93)	246 (93)	107 (94)	
Urinary incontinence (*n* = 185), *n* (%)				
0–1 pad	157 (85)	108 (90)	49 (73)	**0.008**
≥2 pads	28 (15)	12 (10)	16 (27)	
EORTC score, mean (SD); median [range]				
Urinary symptoms (*n* = 361)	28.8 (18.9) 25 [0–83.3]	25.6 (18.2) 20.8 [0–79.2]	36.0 (18.5) 33.3 [0–83.3]	**<0.001**
Bowel symptoms (*n* = 364)	7.5 (12.6) 0 [0–83.3]	6.7 (12.2) 0 [0–75]	9.5 (13.4) 8.3 [0–83.3]	0.054
Hormone symptoms (*n* = 313)	15.3 (13.7) 11.1 [0–72.2]	13.6 (13.4) 11.1 [0–72.2]	19.4 (13.6) 16.7 [0–55.6]	**<0.001**
Sexual activity (*n* = 389)	46.7 (28.6) 50.0 [0–100]	48.7 (28.5) 50 [0–100]	42.0 (28.2) 50 [0–100]	**0.03**
Sexual functioning symptoms (*n* = 215)	47.6 (23.1) 50 [0–100]	43.3 (22.1) 41.7 [0–100]	57.9 (22.1) 58.3 [8.3–100]	**<0.001**

Bold values statistically significant at *P* < 0.05.

In a following multivariate analysis, predictors for a high decision regret were a high score in EORTC urinary symptoms (score ≥25: odds ratio [OR] 2.2, 95% CI 1.1–4.6; *P* = 0.03) and a high score for EORTC sexual functioning symptoms (score ≥50: OR 3.7, 95% CI 1.8–7.4; *P* < 0.01). In contrast to the group‐wise isolated chi‐squared testing, surgical approach (RARP) did not present itself as a significant predictor of decision regret in either a univariate (*P* = 0.3) or multivariate analysis combined with other factors (*P* = 0.3). In the same analysis, PSA recurrence remained a factor with no significant influence on surgical decision regret (*P* = 0.6) (Table 3).

**Table 3:** Logistic regression of predictors for high SDR.

Variable	Univariate		Multivariate	
	OR (95% CI)	*P*	OR (95% CI)	*P*
PSA recurrence (≥0.2 ng/mL)	1.4 (0.4–4.3)	0.57	1.4 (0.4–4.9)	0.57
EORTC urinary symptoms (score ≥25)	2.9 (1.5–5.6)	**<0.01**	2.2 (1.1–4.6)	**0.03**
EORTC sexual functioning symptoms (score ≥50)	4.6 (2.4–9.0)	**<0.01**	3.7 (1.8–7.4)	**<0.01**
Surgical approach (RARP)	0.7 (0.3–1.3)	0.23	0.7 (0.3–1.4)	0.32

Bold values statistically significant at *P* < 0.05.

### The PSM Analyses

The PSM model targeted the average treatment effect in the treated. After matching ORP to RARP cases, 236 patients remained in the matched cohort. Covariate balance improved, with all standardised mean differences <0.1 (age <0.01, D'Amico risk group ≤0.08, household income ≤0.09), indicating successful post‐matching balance between groups. Cohort characteristics are shown in Tables S2 and S3.

Approach‐wise differences in PSA recurrence (*P*_PSM_ = 0.8) and EORTC sexual activity (*P*_PSM_ = 0.1) previously seen in the unmatched cohort, were not observable in the PSM cohort.

Similarly, previously significant differences in continuous surgical decision regret (Table S1) did not appear significant anymore between ORP and RARP in the PSM sample (mean [SD] DRS score 7.4 [13.6] vs 6.9 [13.2], *P*_PSM_ = 0.8). The deviating retrospective assessments of erectile function were comparable among the groups within the PSM sample (*P*_PSM_ = 0.2). Erectile dysfunction remained to be reported as worse than expected for most patients (125.1/221 [57%]) within the PSM cohort (Table S5).

However, grouping by decision regret, functional outcomes differing in the original cohort remained significant within the PSM cohort (Table S3). Matched patients with high surgical decision regret (DRS score >10) showed higher urinary (mean [SD] score 36.0 [16.5] vs 24.8 [16.8], *P*_PSM_ < 0.01), bowel (mean [SD] score 11.0 [12.0] vs 6.1 [11.2], *P*_PSM_ = 0.02), hormone (mean [SD] score 18.9 [13.7] vs 13.4 [12.9], *P*_PSM_ = 0.02) and sexual functioning symptoms (mean [SD] score 60.1 [19.4] vs 44.3 [21.1], *P*_PSM_ < 0.01), as well as lower sexual activity (mean [SD] score 38.3 [26.3] vs 51.0 [27.7], *P*_PSM_ < 0.01). Findings of functional outcomes as main predictors of high surgical decision regret in the multivariate logistic regression model were also confirmed within the PSM cohort (Table S4). Low sexual functioning (score ≥50) remained the strongest predictor for high surgical decision regret (OR 3.4, 95% CI 1.1–10.6; *P*_PSM_ = 0.03), followed by high urinary symptom score (OR 0.3, 95% CI 0.1–0.9; *P*_PSM_ = 0.04).

## Discussion

This large multicentric study updates our understanding of decision regret after ORP and RARP, identifies its drivers and contains valuable information indicating how surgical decision regret can be reduced in clinical practice. High functional symptoms (high EORTC urinary symptoms: OR 2.2, 95% CI 1.1–4.6; *P* = 0.03) and EORTC sexual functioning (OR 3.7, 95% CI 1.8–7.4; *P* < 0.01) are independent predictors for high surgical decision regret, upholding after PSM (OR 3.3, *P*_PSM_ = 0.04 and OR 3.4, *P*_PSM_ = 0.03, respectively), whereas the oncological outcome and the surgical procedure played no role. Postoperative erectile function was the most misjudged factor with 54% of patients having expected a better outcome.

In our sample of 419 patients, the mean (SD) surgical DRS score was 9.0 (15.4) and thereby showing lower values than previously observed in other German and international studies on decision regret after RP [10, 16, 19, 20]. A 2024 meta‐analysis found RP to result in higher decision regret rates compared alternative localised treatment options (radiotherapy, active surveillance or brachytherapy, not statistically tested) [19]. According to an older study from 2020 in Germany [10], no major differences existed among treatment alternatives, and the surgical approach was not an independent predictor for decision regret (*P* = 0.1). This contradiction and the lowered mean decision regret observed in our cohort, calls for newer comparative studies among treatment modalities. Addressing decision regret within RP, the IMPROVE study (German register of clinical studies identifier: DRKS00023765) showed comparable results for ORP and RARP on a higher overall level of regret in 2022 (median DRS score ORP: 15, RARP: 10). However, their data acquisition had a significantly longer follow‐up period for ORP than for RARP [20]. It had previously been shown, how decision regret after RP increases over time [10, 19]. Our data confirms that surgical approach is not a predictor of decision regret.

As previously reported [17], but often challenged, we saw no approach‐dependent EORTC differences in urinary continence (*P* = 0.3), urinary symptoms (*P* = 0.6) or sexual functioning (*P* = 0.6). This was confirmed in the PSM cohort, but especially astonishing considering the higher rate of high‐risk tumours in the ORP group (35% vs 14%, *P* < 0.001). Looking at our multicentre design, this can be explained by the fact that all hospitals are high‐volume centres (Table S6) with high caseload and surgeon expertise, which are required for good outcomes [21, 22].

The outcomes that are perceived as worse than expected are relevant factors in assessing the origins of decision regret. The focus here lies on erectile dysfunction, which more than half the patients found to be worse than expected (54% [221/404]) (Fig. 1). These unmet erectile expectations were higher in patients aged >65 years (113 [57%] vs 108 [52%], *P* = 0.046), whereas age did not differ between groups of high or low surgical decision regret (mean [SD] 64.4 [6.7] vs 65.4 [6.6] years, *P* = 0.2). When looking at postoperative erectile function, it is important to consider two closely related factors defining it: preoperative erectile function and nerve‐sparing surgery used to maintain it. Unfortunately, no information on usage of nerve‐sparing surgery was recorded. However, with 78% of patients being classified as non‐high risk many may have been eligible for nerve‐sparing surgery. Pre‐existing self‐reported problems holding an erection (EORTC Question 23: moderate or very) was prevalent in 43% (114/268) of this cohort, with no differences among the high and low DRS score groups (*P* = 0.6), making it unlikely that pre‐existing problems pre‐defined postoperative regret via postoperative function scores. More comprehensive longitudinal analyses will be needed to clearly rule out causality. However, previous evidence also points to unmatched expectations rather than pre‐existing function as the origin of regret: Wittmann et al. [23] already demonstrated a substantial mismatch between expected and actual postoperative erectile function 1 year after RP, contributing to reduced patient satisfaction. Unmet expectations regarding erectile recovery have also been shown to significantly increase decision regret, particularly when postoperative function is low [24]. This corresponds to our findings, in which poor sexual functioning in the EORTC score is the strongest independent predictor of high decision regret (OR 3.7, *P* < 0.01, PSM: OR 3.4, *P*_PSM_ = 0.03).

Looking at other factors, the oncological outcome plays no role in surgical decision regret (*P* = 0.6, *P*_PSM_ = 0.9) and is rarely rated by patients as worse than expected after surgery (10% [39/397]). However, the low subjective importance of oncological outcome has repeatedly been reported [10, 19, 20]. A British study even showed that patients are willing to trade up to 1% of absolute survival time for better functional outcome with urinary function being slightly more valuable to patients than sexual function [25]. Poor EORTC urinary symptoms (OR 2.2, *P* = 0.03, PSM: OR 3.3, *P*_PSM_ = 0.04) are the second relevant independent predictor for a high decision regret. Nevertheless, fewer patients reported that their urinary situation was worse than expected (23% [96/414]). This can be seen in the context that the overall urinary incontinence rates are lower than those of erectile dysfunction [2, 26].

Ultimately, the question arises as to why erectile dysfunction stands out so prominently – especially in terms of unfulfilled expectations. On one hand, patients may be less objective in their assessment, as issues of masculinity and shame play a role in erectile function [27]. On the other hand, doctors strongly influence how nuanced the postoperative erectile function is discussed. Here, structured interviews of PCa survivor couples revealed that >90% of couples retrospectively overestimated functional, especially erectile, outcomes due to excessive faith in themselves and their surgeons [28]. Shabbir [29] also highlights that the unrealistic expectations of rapid or complete erectile recovery—often due to insufficiently detailed preoperative counselling—play a central role in postoperative disappointment. Even though preoperative counselling was not standardised in the framework of this study, its multicentric design avoids institution‐specific biases.

Several limitations of this study should be acknowledged. First, recall bias may have affected 1‐year retrospective assessments of preoperative expectations, as patients’ recollections of their preoperative expectation levels are likely influenced by their postoperative experiences and outcomes. Second, information on nerve‐sparing status and the use of phosphodiesterase type 5 inhibitors was not included, limiting the ability to further contextualise postoperative sexual outcomes. Patients might think more regretfully about their treatment choice, if they previously held misled hopes in restoring sexual function by surgically removing the cancerous gland. However, no difference in preoperative problems maintaining an erection were observed. Third, despite the multicentric design, all participating institutions were high‐volume centres, which may limit the applicability of results to lower‐volume or non‐specialised settings. Due to the low recurrence rate in this population from highly experienced centres, an even larger population might be needed to evaluate surgical decision regret in patients with cancer recurrence.

This study has notable strengths. Its prospective, multicentre design across seven high‐volume urology centres in Germany and Austria enhances both the internal validity and generalisability of the findings. The inclusion of preoperative and 1‐year postoperative assessments allows for a robust evaluation of decision regret and its determinants. Moreover, the use of validated instruments, including the DRS and the EORTC QLQ‐PR25, ensures reliable measurement of patient‐reported outcomes. Importantly, the simultaneous evaluation of functional outcomes, patient expectations, and surgical approach provides a comprehensive and contemporary perspective on decision regret after RP.

## Conclusion

In this large prospective multicentre cohort, surgical decision regret after RP was driven primarily by postoperative urinary and sexual functional outcomes. Erectile dysfunction was most frequently perceived as worse than expected. These findings underscore the importance of realistic preoperative counselling and expectation management, especially regarding postoperative sexual function. This includes educating especially ORP patients that they are at no measurable disadvantage in terms of postoperative function, contrary to popular belief of RARP as more precise. Optimising functional recovery and aligning patient expectations individually with realistic functional outcomes may represent the most effective strategies to reduce decision regret, balance sexual activity and improve patient satisfaction following PCa surgery.

## Author Contributions

Conceptualisation: Christian Thomas, Martin Baunacke; Methodology: Martin Baunacke, Christopher Hirtsiefer.; Data acquisition: Christopher Hirtsiefer, Emma Kümmel, Anna Vogelgesang, Fabian Falkenbach, Mona Kafka, Annemarie Uhlig, Tim Nestler, Cem Aksoy, Iva Simunovic, Johannes Huber, Isabel Heidegger, Markus Graefen, Marianne Leitsmann, Martin Baunacke; Formal analysis and investigation: Christopher Hirtsiefer; Martin Baunacke; Writing – original draft preparation: Christopher Hirtsiefer; Writing – review and acquisition: Christopher Hirtsiefer, Emma Kümmel, Anna Vogelgesang, Fabian Falkenbach, Mona Kafka, Annemarie Uhlig, Tim Nestler, Cem Aksoy, Iva Simunovic, Johannes Huber, Isabel Heidegger, Markus Graefen, Marianne Leitsmann, Martin Baunacke; Resources: Johannes Huber, Isabel Heidegger, Markus Graefen, Christian Thomas; Supervision: Martin Baunacke. All authors read and approved the final manuscript.

## Funding

The authors declare that no funds, grants, or other support were received for the preparation of this manuscript. Christopher Hirtsiefer receives unrelated funding by the German Research Foundation for a post‐doctoral fellowship at the Vancouver Prostate Centre at the time of preparation of this manuscript. Martin Baunacke received unrelated funding by the German ‘Gemeinsamer Bundesausschuss’ for researching incontinence.

## Disclosure of Interests

Martin Baunacke and Christopher Hirtsiefer have received a one‐time consultancy fee from Medtronic (Minneapolis, MN, USA) in 2025 for services related to robotic systems. All other authors declare no conflicts of interest in relation to the data in this manuscript.

## Ethics Approval

This study was performed in line with the principles of the Declaration of Helsinki. Informed prior written consent was obtained from all patients involved in the study. Approval was granted by the Ethics Committee of the Technical University of Dresden (BO‐EK‐438092021).

## Supporting information


**Appendix S1.** The preoperative questionnaires.

**Appendix S2.** The 1‐year postoperative questionnaires.

**Table S1.** Study sample by surgical method with decision regret and retrospective assessment (*n* = 419).
**Table S2.** The PSM (repeated nearest‐neighbour matching) sample by surgical approach with outcome and QoL (*n* = 236). Values are weighted estimates from the PSM cohort. *N* indicates non‐missing observations. Continuous variables are shown as mean (SD), and median (range), categorical variables as weighted *n* (%). *P* values are based on chi‐squared tests/*t*‐tests of weighted group comparisons with missing values excluded. *Statistical testing not applicable.
**Table S3.** The PSM (repeated nearest‐neighbour matching) sample by surgical decision regret (DRS score) grouping with outcome and QoL (*n* = 229). Continuous variables are shown as *N*, mean (SD), and median (range). Categorical variables are shown as weighted *n* (%). *P* values are based on chi‐squared tests/*t*‐tests of weighted group comparisons with missing values (decision regret values missing for seven in the PSM sample) excluded.
**Table S4.** Weighted logistic regression models in the PSM (repeated nearest‐neighbour matching) sample. The ORs with 95% CIs are shown for high surgical decision regret (DRS score ≥10). Univariate analyses used variable‐specific complete cases; multivariate analysis used complete cases across all included variables.
**Table S5.** The PSM (repeated nearest‐neighbour matching) sample by surgical approach with decision regret and retrospective assessment (*n* = 229). Continuous variables are shown as mean (SD), and median (range). Categorical variables are shown as weighted *n* (%). *P* values are based on weighted *t*‐tests for continuous variables and weighted chi‐squared tests for categorical variables, with missing values excluded.
**Table S6.** Overview of clinical characteristics and key outcomes per centre, including centre count and surgical periods (*n* = 419).

## Data Availability

Original data are available upon request from the corresponding author.
