# Supplementary material for: Function drives 1‐year decision regret in radical prostatectomy: a multicentre prospective study
Source: BJU Int. 2026 Jul 10;138(4):694–704. doi: 10.1111/bju.70382 (PMC13550822; doi:10.1111/bju.70382)
Supplement: Supplementary file 1 [file BJU-138-694-s001.docx]

| **Variable** | | **All**  **(n=419)** | **ORP**  **(n=174)** | **RARP**  **(n=245)** | **p value** |
| --- | --- | --- | --- | --- | --- |
| Therapeutic Decision Regret (N=399) | | 10.0±16.0  0 (0 – 95) | 9.5±15.1  0 (0 – 70) | 10.4±16.6  0 (0 – 95) | *0.6* |
| Surgical Decision Regret (DRS) (N=401) | | 9.0±15.4  0 (0 – 100) | 11.3±17.1  0 (0 – 70) | 7.4±13.9  0 (0 – 100) | ***0.02*** |
| Retrospectively well informed preoperatively (n=416) | | | | | |
|  | Agree | 378 (91%) | 156 (90%) | 222 (91%) | *0.4* |
|  | Partly | 32 (8%) | 15 (8%) | 17 (7%) |  |
|  | Don’t agree | 6 (1%) | 1(1%) | 5 (2%) |  |
| Retrospectively well informed on surgery by experts (n=417) | | | | | |
|  | Agree | 381(91%) | 159 (92%) | 222 (91%) | *0.5* |
|  | Partly | 29 (8%) | 10 (6%) | 19 (8%) |  |
|  | Don’t agree | 7 (2%) | 4 (2%) | 3 (1%) |  |
| Retrospectively wished for better surgical education by experts (n=409) | | | | | |
|  | Agree | 71 (17%) | 27 (16%) | 44 (19%) | *0.6* |
|  | Partly | 35 (9%) | 13 (7%) | 22 (9%) |  |
|  | Don’t agree | 303 (74%) | 130 (77%) | 173 (72%) |  |
| Retrospectively wished for second-opinion (n=393) | | | | | |
|  | Agree | 68 (17%) | 24 (15%) | 44 (19%) | *0.5* |
|  | Partly | 22 (6%) | 9 (5%) | 13 (6%) |  |
|  | Don’t agree | 303 (74%) | 130 (80%) | 173 (75%) |  |
| Would recommend procedure (n=408) | | | | | |
|  | Agree | 336 (83%) | 132 (78%) | 204 (86%) | *0.06* |
|  | Partly | 42 10%) | 20 (12%) | 22 (9%) |  |
|  | Don’t agree | 30 (7%) | 18 (10%) | 12 (5%) |  |
| Urinary incontinence (n=414) | | | | | |
|  | Better | 210 (51%) | 87 (51%) | 123 (51%) | *1.0* |
|  | As expected | 108 (26%) | 45 (26%) | 63 (26%) |  |
|  | Worse | 96 (23%) | 39 (23%) | 57 (23%) |  |
| Erectile dysfunction (n=404) | | | | | |
|  | Better | 47 (12%) | 9 (5%) | 38 (16%) | ***0.004*** |
|  | As expected | 136 (34%) | 59 (35%) | 77 (33%) |  |
|  | Worse | 221 (54%) | 100 (60%) | 121 (51%) |  |
| Oncologic cure (n=397) | | | | | |
|  | Better | 187 (47%) | 71 (44%) | 116 (49%) | *0.6* |
|  | As expected | 171 (43%) | 73 (45%) | 98 (42%) |  |
|  | Worse | 39 (10%) | 17 (11%) | 22 (9%) |  |
| Complications (n=400) | | | | | |
|  | Better | 224 (56%) | 89 (55%) | 135 (57%) | *0.8* |
|  | As expected | 133 (33%) | 58 (35%) | 75 (32%) |  |
|  | Worse | 43 (11%) | 17 (10%) | 26 (11%) |  |
| Wound healing (n=414) | | | | | |
|  | Better | 254 (61%) | 108 (63%) | 146 (60%) | *0.8* |
|  | As expected | 122 (29%) | 48 (28%) | 74 (31%) |  |
|  | Worse | 38 (10%) | 16 (9%) | 22 (9%) |  |
| Duration of hospital stay (n=413) | | | | | |
|  | Better | 163 (39%) | 59 (35%) | 104 (43%) | ***0.02*** |
|  | As expected | 220 (54%) | 93 (54%) | 127 (52%) |  |
|  | Worse | 30 (7%) | 19 (11%) | 11 (5%) |  |
| Duration of catherization (n=407) | | | | | |
|  | Better | 111 (27%) | 36 (21%) | 75 (31%) | *0.06* |
|  | As expected | 199 (49%) | 85 (51%) | 114 (48%) |  |
|  | Worse | 97 (24%) | 47 (28%) | 50 (21%) |  |
| Time of convalescence (n=410) | | | | | |
|  | Better | 148 (36%) | 57 (34%) | 91 (38%) | *0.5* |
|  | As expected | 169 (41%) | 70 (41%) | 99 (41%) |  |
|  | Worse | 93 (23%) | 43 (25%) | 50 (21%) |  |

**Supplementary Table 1:** Study Sample by Surgical Method with decision regret and retrospective assessment (n=419).

| **Variable** | | **All**  **(n=236)** | **ORP**  **(n=47)** | **RARP**  **(n=189)** | **p value** |
| --- | --- | --- | --- | --- | --- |
| Age at surgery | | 64.3 ± 6.8  65 (45–78) | 64.3 ± 6.4  67 (53–77) | 64.3 ± 6.9  65 (45–78) | *1.0* |
| D’Amico Risk Group (n=236) | | | | | |
|  | Low | 47.7 (20%) | 10.7 (23%) | 37 (20%) | *0.9* |
|  | Intermediate | 162.8 (69%) | 30.8 (66%) | 131 (69%) |  |
|  | High | 26.5 (11%) | 5.5 (12%) | 21 (11%) |  |
| Insurance (n=235) | | | | | |
|  | Statutory | 155.1 (66%) | 37.1 (79%) | 118 (63%) | *0.09* |
|  | Private | 79.9 (34%) | 9.9 (21%) | 70 (37%) |  |
| Residential Area (n=233) | | | | | |
|  | Rural | 100.9 (43%) | 15.9 (34%) | 85 (46%) | *0.3* |
|  | Urban | 132.1 (57%) | 31.1 (66%) | 101 (54%) |  |
| Marital Status (n=233) | | | | | |
|  | Single | 19 (8%) | 2.7 (6%) | 16 (9%) | *0.6* |
|  | Married | 215 (92%) | 44 (94%) | 171 (91%) |  |
| Education (n=217) | | | | | |
|  | High School (Hauptschule) | 44.7 (21%) | 8.7 (20%) | 36 (21%) | *0.5* |
|  | High School (Realschule) | 73.9 (34%) | 18.9 (43%) | 55 (32%) |  |
|  | Baccalaureate | 99.4 (46%) | 16.4 (37%) | 83 (48%) |  |
| Monthly household income (EUR) (n=236) | | | | | |
|  | <1500 | 12.2 (5%) | 2.2 (5%) | 10 (5%) | *0.9* |
|  | 1500-4000 | 124.4 (53%) | 23.4 (50%) | 101 (54%) |  |
|  | >4000 | 99.4 (42%) | 21.4 (45%) | 78 (41%) |  |
| Centre Distribution (n=236) | | | | | |
|  | Dresden | 100.2 (42%) | 18.2 (39%) | 82 (43%) | ******* |
|  | Hamburg | 77.4 (33%) | 20.4 (43%) | 57 (30%) |  |
|  | Marburg | 31.7 (13%) | 3.7 (8%) | 28 (15%) |  |
|  | Graz | 26.7 (11%) | 4.7 (10%) | 22 (12%) |  |
| PSA recurrence 0.2+ ng/ml (n=223) | | | | | |
|  | Yes | 7.7 (3.5%) | 1.7 (4%) | 6 (3%) | *0.8* |
|  | No | 213.5 (96.5%) | 40.5 (96%) | 173 (97%) |  |
| Urinary incontinence (n=105) | | | | | |
|  | Yes | 11 (10%) | 0 (0%) | 11 (13%) | *0.08* |
|  | No | 94.9 (90%) | 22.9 (100%) | 72 (87%) |  |
| EORTC Score | | | | | |
|  | Urinary Symptoms (n=214) | 27.5 ± 17.4  25.0 (0–79.2) | 25.4 ± 14.1  25.0 (0–54.2) | 28.1 ± 18.1  25.0 (0–79.2) | 0.4 |
|  | Bowel Symptoms (n=215) | 7.3 ± 11.5  0 (0–66.7) | 8.0 ± 10.1  0 (0–41.7) | 7.2 ± 11.8  0 (0–66.7) | 0.7 |
|  | Hormone Symptoms (n=188) | 14.7 ± 13.2  11.1 (0–72.2) | 15.7 ± 12.1  11.1 (0–55.5) | 14.5 ± 13.5  11.1 (0–72.2) | 0.6 |
|  | Sexual Activity (n=231) | 47.4 ± 27.6  50.0 (0–100.0) | 39.6 ± 27.9  33.3 (0–83.3) | 49.4 ± 27.2  50.0 (0–100.0) | 0.12 |
|  | Sexual Functioning Symptoms (n=129) | 48.1 ± 21.5  41.7 (0–100.0) | 56.2 ± 16.9  58.3 (16.7–75.0) | 46.7 ± 22.0  41.7 (0–100.0) | 0.08 |

**Supplementary Table 2:** Propensity-Score matched (PSM, repeated nearest neighbour matching) sample by surgical approach with outcome and quality of life (n=236). Values are weighted estimates from the propensity score matched cohort. N indicates non-missing observations. Continuous variables are shown as mean ± SD, and median (min–max), categorical variables as weighted n (%). P values are based on Chi2-tests/t-tests of weighted group comparisons with missing values excluded. * Statistical testing not applicable.

| **Variable** | | | **All**  **(n= 229)** | **Low surgical DR (<10)**  **(n=165)** | **High surgical DR (10+)**  **(n=64)** | **p value** |
| --- | --- | --- | --- | --- | --- | --- |
| Age at surgery | | | 64.2 ± 6.8  65.0 (45–78) | 63.9 ± 7.0  64.0 (45–78) | 65.1 ± 6.2  67.0 (51–78) | *0.2* |
| D’Amico Risk Group (n=229) | | |  |  |  |  |
|  | Low | | 44.7 (19%) | 34.5 (21%) | 10.2 (17%) | *0.6* |
|  | Intermediate | | 159.8 (70%) | 117.1 (70%) | 42.7 (69%) |  |
|  | High | | 25.2 (11%) | 16.5 (10%) | 8.7 (14%) |  |
| Insurance (n=228) | | |  |  |  |  |
|  | Statutory | | 151.8 (66%) | 109.1 (65%) | 42.7 (69%) | *0.6* |
|  | Private | | 76.9 (34%) | 58 (35%) | 19 (31%) |  |
| Residential Area (n=226) | | |  |  |  |  |
|  | Rural | | 97.9 (43%) | 68.2 (41%) | 29.7 (48%) | *0.4* |
|  | Urban | | 128.8 (57%) | 96.9 (59%) | 32 (52%) |  |
| Marital Status (n=226) | | |  |  |  |  |
|  | Single | | 18.7 (8%) | 10.7 (6%) | 8 (13%) | *0.12* |
|  | Married | | 208.8 (92%) | 155.1 (94%) | 53.7 (87%) |  |
| Education (n=210) | | |  |  |  |  |
|  | High School (Hauptschule) | | 44.7 (21%) | 25 (16%) | 19.7 (34%) | ***0.03*** |
|  | High School (Realschule) | | 71.7 (34%) | 54.4 (35%) | 17.2 (30%) |  |
|  | Baccalaureate | | 95.4 (45%) | 74.9 (49%) | 20.5 (36%) |  |
| Monthly household income (n=229) | | |  |  |  |  |
|  | <1500 € | | 12.2 (5%) | 8.5 (5%) | 3.7 (6%) | *0.8* |
|  | 1500-4000 € | | 120.1 (52%) | 86.4 (51%) | 33.7 (55%) |  |
|  | >4000 € | | 97.4 (42%) | 73.2 (44%) | 24.2 (39%) |  |
| Second opinion used (n=225) | | |  |  |  |  |
|  | Yes | | 114.4 (51%) | 82.9 (50%) | 31.5 (52%) | *0.8* |
|  | No | | 111.4 (49%) | 82.2 (50%) | 29.2 (48%) |  |
| Decisional behavior (n=222) | | |  |  |  |  |
|  | Autonomous | | 71.4 (32%) | 53.4 (33%) | 18 (29%) | *0.6* |
|  | Shared | | 140.1 (63%) | 98.7 (61%) | 41.4 (67%) |  |
|  | Paternalistic | | 11.5 (5%) | 9.2 (6%) | 2.2 (4%) |  |
| Surgical method (n=229) | | |  |  |  |  |
|  | ORP | | 46.8 (20%) | 31.1 (18%) | 15.7 (25%) | *0.4* |
|  | RARP | | 183 (80%) | 137 (82%) | 46 (75%) |  |
| Surgical methods offered (n=229) | | |  |  |  |  |
|  | Both | | 229.8 (100%) | 168.1 (100%) | 61.7 (100%) | * |
| PSA recurrence ≥0.2 ng/ml (n=217) | | | | | | |
|  | Yes | 7.7 (4%) | | 5.5 (3%) | 2.2 (4%) | *0.9* |
|  | No | 208.3 (96%) | | 152.9 (97%) | 55.4 (96%) |  |
| Urinary incontinence (n=104) | |  | |  |  |  |
|  | 0-1 pad | 94.9 (90%) | | 63.9 (91%) | 30.9 (89%) | *0.6* |
|  | ≥2 pads | 10 (10%) | | 6 (9%) | 4 (11%) |  |
| EORTC Score | |  | |  |  |  |
|  | Urinary Symptoms (n=208) | 27.9 ± 17.4  25.0 (0–79.2) | | 24.8 ± 16.8  20.8 (0–70.8) | 36.0 ± 16.5  33.3 (4.2–79.2) | ***<0.01*** |
|  | Bowel Symptoms (n=209) | 7.5 ± 11.6  0.0 (0–66.7) | | 6.1 ± 11.1  0.0 (0–66.7) | 11.0 ± 12.0  8.3 (0–41.7) | ***0.02*** |
|  | Hormone Symptoms (n=183) | 14.8 ± 13.3  11.1 (0–72.2) | | 13.4 ± 12.9  11.1 (0–72.2) | 18.9 ± 13.7  16.7 (0–55.6) | ***0.02*** |
|  | Sexual Activity (n=224) | 47.6 ± 27.9  50.0 (0–100.0) | | 51.0 ± 27.7  50.0 (0–100.0) | 38.3 ± 26.3  50.0 (0–100.0) | ***<0.01*** |
|  | Sexual Functioning Symptoms (n=125) | 47.9 ± 21.7  41.7 (0–100.0) | | 44.3 ± 21.1  41.7 (0–100.0) | 60.1 ± 19.4  58.3 (16.7–100.0) | ***<0.01*** |

**Supplementary Table 3:** Propensity-Score matched (PSM, repeated nearest neighbour matching) sample by surgical decision regret (DRS) grouping with outcome and quality of life (n=229). Continuous variables are shown as N, mean ± SD, and median (min–max). Categorical variables are shown as weighted n (%). P values are based on Chi2-tests/t-tests of weighted group comparisons with missing values (Decision Regret missing for N=7 in PSM sample) excluded.

| Variable | Univariate | | | Multivariate | | |
| --- | --- | --- | --- | --- | --- | --- |
|  | N | OR (95% CI) | p value | N | OR (95% CI) | p value |
| PSA recurrence (0.2+ ng/ml) | 217 | 1.1 (0.2–5.5) | 0.9 | 111 | 2.5 (0.3–21.7) | 0.4 |
| EORTC Urinary Symptoms (score≥25) | 208 | 3.3 (2.0–10.0) | **<0.01** | 111 | 3.3 (1.1–10.0) | **0.04** |
| EORTC Sexual Functioning Symptoms (score≥50) | 125 | 4.1 (1.6–10.7) | **<0.01** | 111 | 3.4 (1.1–10.6) | **0.03** |
| Surgical Approach (RARP) | 229 | 0.7 (0.3–1.6) | 0.4 | 111 | 0.8 (0.1–6.8) | 0.9 |

**Supplementary Table 4:** Weighted logistic regression models in the Propensity-Score matched (PSM, repeated nearest neighbour matching) sample. Odds ratios (OR) with 95% confidence intervals (CI) are shown for high Surgical Decision Regret (DRS 10+). Univariate analyses used variable-specific complete cases; multivariate analysis used complete cases across all included variables.

| **Variable** | | **All**  **(n=229)** | **ORP**  **(n=46)** | **RARP**  **(n=183)** | **p value** |
| --- | --- | --- | --- | --- | --- |
| Therapeutic Decision Regret (N=227) | | 9.0 ± 14.8  0 (0–95.0) | 8.6 ± 12.7  0 (0–55.0) | 9.1 ± 15.3  0 (0–95.0) | *0.9* |
| Surgical Decision Regret (DRS) (N=229) | | 7.0 ± 13.2  0 (0–100.0) | 7.4 ± 13.6  0 (0–60.0) | 6.9 ± 13.2  0 (0–100.0) | *0.8* |
| Erectile dysfunction (n=221) | | | | | |
|  | Better | 25.5 (12%) | 1.5 (3%) | 24 (14%) | *0.2* |
|  | As expected | 70.2 (32%) | 13.2 (29%) | 57 (32%) |  |
|  | Worse | 125.1 (57%) | 30.1 (67%) | 95 (54%) |  |
| Duration of hospital stay (n=229) | | | | | |
|  | Better | 95.7 (42%) | 17.7 (38%) | 78 (43%) | *0.2* |
|  | As expected | 114.4 (50%) | 21.4 (46%) | 93 (51%) |  |
|  | Worse | 18.7 (8%) | 7.7 (16%) | 11 (6%) |  |

**Supplementary Table 5:** Propensity-Score matched (PSM, repeated nearest neighbour matching) sample by surgical approach with decision regret and retrospective assessment (n=229). Continuous variables are shown as mean ± SD, and median (min–max). Categorical variables are shown as weighted n (%). P values are based on weighted t-tests for continuous variables and weighted chi-squared tests for categorical variables, with missing values excluded.

| **Variable** | | **All** (n=419) 1/2022 – 11/2023 | **Centre 1** (n=132) 1/22 – 10/22 | **Centre 2** (n=43) 06/22 – 08/22 | **Centre 3** (n=43**)** 09/22 – 02/23 | **Centre 4** (n=80) 03/22 – 06/22 | **Centre 5** (n=44) 06/22 – 03/23 | **Centre 6** (n=44) 01/22 – 06/23 | **Centre 7** (n=33) 12/22 – 12/23 | |
| --- | --- | --- | --- | --- | --- | --- | --- | --- | --- | --- |
| Age | | 64.8 ± 6.7 65.0 (45.0 – 78.0) | 66.0 ± 6.5 66.5 (48.0 – 78.0) | 66.9 ± 6.4 68.0 (53.0 – 78.0) | 63.8 ± 7.5 64.0 (47.0 – 77.0) | 63.4 ± 6.8 64.0 (45.0 – 76.0) | 64.6 ± 6.2 65.5 (53.0 – 78.0) | 65.7 ± 6.4 66.0 (54.0 – 76.0) | 61.5 ± 6.3 62.0 (46.0 – 71.0) | |
| D’Amico Risk Group | | | | | | | | | |  |
|  | low risk | 70 (17%) | 19 (14%) | 5 (12%) | 8 (19%) | 18 (22%) | 11 (25%) | 4 (9%) | 5 (17%) | |
|  | intermediate risk | 250 (60%) | 79 (60%) | 27 (64%) | 26 (60%) | 49 (61%) | 24 (55%) | 23 (53%) | 22 (73%) | |
|  | high risk | 94 (23%) | 34 (26%) | 10 (24%) | 9 (21%) | 13 (16%) | 9 (20%) | 16 (37%) | 3 (10%) | |
| Monthly household income (EUR) | | | | | | | | | |  |
|  | <1500 | 27 (7%) | 13 (12%) | 1 (3%) | 2 (5%) | 3 (4%) | 4 (11%) | 3 (7%) | 1 (3%) | |
|  | 1500-4000 | 216 (57%) | 68 (61%) | 24 (63%) | 26 (67%) | 24 (31%) | 29 (76%) | 25 (61%) | 20 (65%) | |
|  | >4000 | 133 (35%) | 31 (28%) | 13 (34%) | 11 (28%) | 50 (65%) | 5 (13%) | 13 (32%) | 10 (32%) | |
| Surgical approach (RARP) | | 256 (61%) | 96 (73%) | 43 (100%) | 0 (0%) | 59 (74%) | 33 (75%) | 0 (0%) | 25 (76%) | |
|  | | **Outcome parameters** | | | | | | | |  |
| Surgical Decision Regret | | | | | | | | | |  |
|  | Low (<10) | 277 (69%) | 88 (69%) | 32 (78%) | 27 (66%) | 60 (78%) | 25 (57%) | 24 (60%) | 21 (68%) | |
|  | High (10+) | 124 (31%) | 39 (31%) | 9 (22%) | 14 (34%) | 17 (22%) | 19 (43%) | 16 (40%) | 10 (32%) | |
| PSA recurrence 0.2+ ng/ml | | 26 (7%) | 3 (2%) | 5 (12%) | 4 (10%) | 5 (6%) | 3 (8%) | 5 (12%) | 1 (3%) | |
| Urinary Incontinence (≥2 pads) | | 32 (17%) | 12 (15%) | 1 (5%) | 2 (14%) | 2 (9%) | 7 (33%) | 7 (32%) | 1 (8%) | |
| EORTC Urinary Symptoms (score ≥25) | | 167 (45%) | 63 (51%) | 16 (40%) | 15 (38%) | 28 (39%) | 16 (46%) | 15 (44%) | 14 (44%) | |
| EORTC Sexual Functioning Symptoms (score ≥50) | | 88 (40%) | 27 (50%) | 6 (27%) | 11 (46%) | 17 (31%) | 9 (60%) | 9 (39%) | 9 (32%) | |

**Supplementary Table 6:** Overview of clinical characteristics and key outcomes per centre, including centre count and surgical periods (n=419)
