# Supplementary material for: Function drives 1‐year decision regret in radical prostatectomy: a multicentre prospective study
Source: BJU Int. 2026 Jul 10;138(4):694–704. doi: 10.1111/bju.70382 (PMC13550822; doi:10.1111/bju.70382)
Supplement: Supplementary file 2 [file BJU-138-694-s002.docx]

**ARZT	ID: GR __ __ __**

1. Alter des Patienten:	_____ Jahre	**OP-Datum:**	____________
2. PSA-Wert:	_____ ng/ml
3. Gleason-Score:
   - Gleason 6
   - Gleason 7a
   - Gleason 7b
   - Gleason 8
   - Gleason 9
   - Gleason 10
4. T-Stadium:
   - cT1
   - cT2
   - cT3
   - cT4
5. N-Stadium:
   - cN0
   - cN1
   - cNx
6. M-Stadium:
   - cM0
   - cM1
   - cMx
7. Geplantes OP-Verfahren:
   - offen
   - roboter-assistiert

**PATIENT	ID: GR __ __ __**

1. Wie gut sind Ihre Deutschkenntnisse?
   - Muttersprache
   - Fließend
   - Gering
2. Wie viele Einwohner hat Ihr Wohnort?

- Mehr als 10.000
- Weniger als 10.000

3. Wie sind Sie krankenversichert? (nur Hauptabsicherung unabhängig von möglicher Beihilfe)
   - Gesetzlich
   - Privat
   - Sonstige
4. Wie ist Ihr derzeitiger Familienstand?
   - Alleinstehend (z.B. geschieden, verwitwet)
   - Verheiratet oder in fester Partnerschaft
   - Sonstiges
5. Welchen höchsten Schulabschluss haben Sie?
   - Keinen Abschluss
   - Hauptschulabschluss
   - Realschulabschluss oder Polytechnische Oberschule
   - (Fach-) Abitur
   - Sonstiges
6. Wie hoch ist Ihr monatliches Netto-Haushaltseinkommen (Einkommen aller Haushaltsmitglieder abzüglich Steuern und Abgaben)? Bitte schätzen Sie den Betrag.
   - Weniger als 1.500 €
   - 1.500 – 4.000 €
   - Mehr als 4.000 €
   - Keine Angabe
7. Mein/e behandelnde/r Urologe/in ist
   - Weiblich
   - Männlich
8. Wie alt ist ihr/e behandelnde/r Urologe/in? Schätzen Sie bitte das Alter.
   - 30 – 45 Jahre
   - 46 – 55 Jahre
   - 56 – 65 Jahre
   - Älter als 65 Jahre
9. Wo arbeitet ihr/e behandelnde/r Urologe/in?
   - Allein in einer Einzelpraxis
   - Zusammen mit weiteren Urologen in einer Gemeinschaftspraxis
   - In einem Krankenhaus

**Informations- und Entscheidungsverhalten**

In den folgenden drei Fragen geht es darum, wie stark Sie selbst an Entscheidungen teilnehmen möchten. Die Antworten sind stets identisch und reichen von einem sehr starken eigenen Anteil (selbständig treffen) bis hin zu einer vollständigen Überlassung an den Arzt (meinem Arzt allein überlassen).

10. Wie möchten Sie medizinische Entscheidungen treffen, die ihre Gesundheit betreffen?

Die Behandlungsentscheidung möchte ich...

- selbständig treffen.
- selbst treffen, aber die Meinung meines Arztes einbeziehen.
- mit meinem Arzt gemeinsam treffen.
- meinem Arzt überlassen, der meine Meinung einbezieht.
- meinem Arzt allein überlassen.

11. Wer hat die Entscheidung getroffen, welches OP-Verfahren zur Behandlung Ihres Prostatakrebses angewandt werden soll? Das heißt ob offen oder mithilfe eines OP-Roboters (DaVinci®) vorgegangen werden soll.

Die Behandlungsentscheidung habe ich...

- selbständig getroffen.
- selbst getroffen, aber die Meinung meines Arztes einbezogen.
- mit meinem Arzt gemeinsam getroffen.
- meinem Arzt überlassen, der meine Meinung einbezogen hat.
- meinem Arzt allein überlassen.

12. Wie wurde das behandelnde Krankenhaus ausgewählt?

Die Wahl des Krankenhauses, habe ich ...

- selbständig getroffen.
- selbst getroffen, aber die Meinung meines Arztes einbezogen.
- mit meinem Arzt gemeinsam getroffen.
- meinem Arzt überlassen, der meine Meinung einbezogen hat.
- meinem Arzt allein überlassen.

13. Über wem haben Sie sich über Operationsverfahren zur Behandlung des Prostatakarzinoms informiert und wie wichtig war diese Informationsquelle für Sie? (Mehrere Antworten möglich)

|  | Keine Information | Überhaupt nicht wichtig | Nicht wichtig | Weder noch | wichtig | Sehr wichtig |
| --- | --- | --- | --- | --- | --- | --- |
| Mein/e behandelnde/r Urologe/in |  |  |  |  |  |  |
| Hausarzt oder anderer Arzt |  |  |  |  |  |  |
| Die Klinik in der meine Operation stattfindet |  |  |  |  |  |  |
| Zweitmeinung |  |  |  |  |  |  |
| Selbsthilfegruppe |  |  |  |  |  |  |
| Mein/e Partner/in |  |  |  |  |  |  |
| Angehörige/Freunde |  |  |  |  |  |  |
| Internet |  |  |  |  |  |  |
| Fernsehen/Radio |  |  |  |  |  |  |
| Broschüren/Zeitschriften |  |  |  |  |  |  |

14. Wie häufig nutzen Sie das Internet zur Informationssuche bei Gesundheitsfragen?
    - Täglich
    - Mindestens 1-mal pro Woche
    - Seltener
    - Gar nicht
15. Welche Internetquellen haben Sie zur Informationssuche bezüglich Ihrer Prostatakarzinomerkrankung genutzt? (Mehrere Antworten möglich)
    - Internetseiten von medizinischen Fachgesellschaften
    - Internetseiten von Zeitungen/Zeitschriften
    - Video-Plattformen (z.B. Youtube)
    - Social Media-Seiten (z.B. Facebook, Instagramm)
    - Online-Selbsthilfe-Foren
    - Wikipedia
    - andere
    - keine
16. Wurde Ihnen zur Frage der Behandlung des Prostatakarzinoms eine Zweitmeinung angeboten?
    - Ja
    - Nein
17. Haben sie zur Frage der Behandlung des Prostatakarzinoms eine Zweitmeinung in Anspruch genommen?
    - Ja
    - Nein
18. Bei Ihrer Informationssuche und Gesprächen über operative Verfahren zur Entfernung der Prostata…
    - …erhielt ich überwiegend Informationen zur Operation mit dem Roboter.
    - …erhielt ich etwa zu gleichen Teilen Informationen zur Operation mit dem Roboter und offenen Operation.
    - …erhielt ich überwiegend Informationen zur offenen Operation.
19. Wie wichtig ist es für sie…

| Wie wichtig ist es für sie… | Überhaupt nicht wichtig… …extrem wichtig | | | | | | | | | |  |
| --- | --- | --- | --- | --- | --- | --- | --- | --- | --- | --- | --- |
|  | 1 | 2 | 3 | 4 | 5 | 6 | 7 | 8 | 9 | 10 | |
| …den Prostatakrebs unmittelbar wirksam zu behandeln? |  |  |  |  |  |  |  |  |  |  | |
| …ungewollten Urinverlust zu vermeiden? |  |  |  |  |  |  |  |  |  |  | |
| …sexuelle Probleme zu vermeiden? |  |  |  |  |  |  |  |  |  |  | |
| …das modernste Therapieverfahren zu bekommen? |  |  |  |  |  |  |  |  |  |  | |
| …operative Risiken zu vermeiden. |  |  |  |  |  |  |  |  |  |  | |

20. Wurden Sie in der Vergangenheit operiert und mit welchen Verfahren?
    - Offene Operation
    - Herkömmliche „Schlüsselloch“-Operation (laparoskopisch)
    - Minimalinvasive Operation mit dem Roboter (DaVinci®)
    - Ich wurde noch nie operiert
21. Wie beurteilen Sie die folgenden Punkte in Abhängigkeit des operativen Verfahrens (offene Operation vs. Operation mit dem Roboter) aufgrund ihrer Informationen, die Sie bisher erhalten haben.

|  | Offene Operation Operation mit dem  Roboter | | | | | Ich habe hierzu keine Meinung |
| --- | --- | --- | --- | --- | --- | --- |
|  | besser | Leicht besser | gleich | Leicht besser | besser |  |
| Risiko der Harninkontinenz |  |  |  |  |  |  |
| Risiko der Erektionsstörung |  |  |  |  |  |  |
| Risiko einer unvollständigen Krebserkrankung |  |  |  |  |  |  |
| Risiko für Komplikationen |  |  |  |  |  |  |
| Schmerzen nach der Operation |  |  |  |  |  |  |
| Wundheilung |  |  |  |  |  |  |
| Dauer des Krankenhausaufenthaltes |  |  |  |  |  |  |
| Dauer des einliegenden Harnblasenkatheter |  |  |  |  |  |  |
| Zeit bis zur vollen Genesung |  |  |  |  |  |  |
| Erfahrung der behandelnden Klinik |  |  |  |  |  |  |

**Lebensqualität**

Patienten klagen oft über die folgenden Symptome und Probleme. Geben Sie bitte an, in welchem

Umfang diese Symptome und Probleme während der letzten Woche bei Ihnen aufgetreten sind.

Markieren Sie bitte die Zahl, die Ihrem Fall am ehesten entspricht.

**Während der letzten Woche...**

**Überhaupt	Wenig	Mäßig	Sehr**

**nicht**

1. Mussten Sie **tagsüber** häufig Wasser lassen?...............................	①	②	③	④

2. Mussten Sie **nachts** häufig Wasser lassen?...................................	①	②	③	④

3. Wenn Sie Harndrang verspürten, mussten Sie sich

dann beeilen, auf die Toilette zu kommen? ………………………..	①	②	③	④

4. Hatten Sie Schwierigkeiten, genug Schlaf zu bekommen, weil

Sie nachts häufig aufstehen mussten, um Wasser zu lassen? ……...	①	②	③	④

5. War es schwierig für Sie, aus dem Haus zu gehen,

weil Sie in der Nähe einer Toilette bleiben mussten? ……………...	①	②	③	④

6. Kam es bei Ihnen zu unwillkürlichem Harnabgang? ……………	①	②	③	④

7. Hatten Sie Schmerzen beim Wasserlassen? ……………………..	①	②	③	④

8. Beantworten Sie diese Frage bitte nur, wenn Sie eine

Inkontinenzhilfe tragen: Empfanden Sie es als Problem,

eine Inkontinenzhilfe zu tragen? …………………………………..	①	②	③	④

9. Wurden Sie in Ihren täglichen Aktivitäten durch Ihre

Blasenprobleme eingeschränkt? ……………………………………	①	②	③	④

10. Wurden Sie in Ihren täglichen Aktivitäten durch Ihre

Darmprobleme eingeschränkt? …………………………………….	①	②	③	④

11. Kam es bei Ihnen zu unwillkürlichem Abgang von Stuhl? ……	①	②	③	④

12. Hatten Sie Blut im Stuhl? ……………………………………...	①	②	③	④

13. Hatten Sie das Gefühl, dass Ihr Bauch aufgebläht ist? ………..	①	②	③	④

14. Hatten Sie Hitzewallungen? ……………………………………	①	②	③	④

15. Hatten Sie schmerzende oder vergrößerte

Brustdrüsen oder Brustwarzen? ……………………………………	①	②	③	④

16. Hatten Sie Schwellungen in Ihren Beinen oder Knöcheln? ……	①	②	③	④

**Während der letzten 4 Wochen...**

**Überhaupt	Wenig	Mäßig	Sehr**

**nicht**

17. Empfanden Sie die Gewichts**abnahme** als Problem? …………	①	②	③	④

18. Empfanden Sie die Gewichts**zunahme** als Problem? ………….	①	②	③	④

19. Haben Sie sich aufgrund Ihrer Krankheit oder der

Behandlung weniger männlich gefühlt? …………………………...	①	②	③	④

20. Hatten Sie Interesse an Sex? …………………………………...	①	②	③	④

21. Waren Sie sexuell aktiv (mit oder ohne Geschlechtsverkehr)? .. ①	②	③	④

**BEANTWORTEN SIE DIE NÄCHSTEN VIER FRAGEN BITTE NUR, WENN SIE WÄHREND DER LETZTEN 4 WOCHEN SEXUELL AKTIV WAREN**

**Überhaupt	Wenig	Mäßig	Sehr**

**nicht**

22. Konnten Sie Sex genießen? …………………………………….. ①	②	③	④

23. Hatten Sie Schwierigkeiten, eine Erektion zu

bekommen oder aufrechtzuerhalten? ……………………………..... ①	②	③	④

24. Hatten Sie Probleme mit dem Samenerguss

(z.B. trockene Ejakulation)? ……………………………………….	①	②	③	④

25. War Ihnen bei sexueller Intimität oder dem

Gedanken an sexuelle Intimität unwohl? …………………………..	①	②	③	④

26. Wie viele Einlagen oder Inkontinenzvorlagen haben Sie normalerweise in den letzten 4 Wochen am Tag gebraucht, um den Urinverlust unter Kontrolle zu haben?

- Keine
- Eine Einlage am Tag
- Zwei Einlagen am Tag
- Drei oder mehr Einlagen am Tag
