# Supplementary material for: Function drives 1‐year decision regret in radical prostatectomy: a multicentre prospective study
Source: BJU Int. 2026 Jul 10;138(4):694–704. doi: 10.1111/bju.70382 (PMC13550822; doi:10.1111/bju.70382)
Supplement: Supplementary file 3 [file BJU-138-694-s003.docx]

**PATIENT	ID: GR __ __ __**

1. Welches OP-Verfahren haben Sie erhalten?
   - Offene Prostataentfernung (Bauchschnitt)
   - Robotisch-assistierte minimal-invasive Operation (DaVinci®)
2. Wann wurde das letzte Mal der PSA-Wert bei Ihnen bestimmt:	_________
3. Wie hoch war der PSA-Wert bei der letzten Bestimmung:	_________ng/ml

 nicht nachweisbar

4. Erhielten oder erhalten Sie eine Hormonbehandlung?
   - Ja, aber aktuell erhalte ich keine Hormonbehandlung mehr
   - Ja, ich erhalte aktuell eine Hormonbehandlung
   - Nein
   - Weiß nicht
5. Falls sie aktuelle eine Hormonbehandlung erhalten, wie ist diese langfristig geplant?
   - Sie soll dauerhaft erfolgen, Pausen sind nicht vorgesehen
   - Sie soll mit Unterbrechungen durchgeführt werden, Pausen sind vorgesehen
   - Sie erfolgt als Vorbereitung auf eine örtliche Behandlung wie eine Operation oder Bestrahlung
   - Weiß nicht
6. Waren die Schnittränder in der feingeweblichen Untersuchung frei oder fanden sich dort Prostatakrebszellen?
   - Die Prostata wurde im Gesunden entfernt (R0)
   - Am Schnittrand fanden sich (fraglich) Prostatakrebszellen (R1 oder Rx)
   - Weiß nicht
7. Bitte geben Sie zu jeder Aussage an, welche Antwort am ehesten auf Sie persönlich zutrifft.

|  | Trifft vollständig zu | Trifft eher zu | Trifft teilweise zu | Trifft eher nicht zu | Trifft überhaupt nicht zu |
| --- | --- | --- | --- | --- | --- |
| Rückblickend war ich über den operativen Eingriff gut informiert |  |  |  |  |  |
| Rückblickend wurde ich von den Ärzten über den operativen Eingriff gut informiert |  |  |  |  |  |
| Rückblickend hätte ich mir mehr Informationen über den operativen Eingriff gewünscht |  |  |  |  |  |
| Rückblickend hätte ich mir eine Zweitmeinung eingeholt |  |  |  |  |  |
| Den operativen Eingriff würde ich betroffenen Freunden oder Verwandten empfehlen |  |  |  |  |  |

8. Wie beurteilen Sie rückblickend Ihre Erwartungshaltung zu folgenden Aspekten der Operation?

|  | deutlich besser als erwartet | besser als erwartet | wie erwartet | schlechter als erwartet | deutlich schlechter als erwartet |
| --- | --- | --- | --- | --- | --- |
| Risiko Harninkontinenz |  |  |  |  |  |
| Risiko Erektionsstörung |  |  |  |  |  |
| Risiko einer unvollständigen Krebsentfernung |  |  |  |  |  |
| Risiko für Komplikationen |  |  |  |  |  |
| Wundheilung |  |  |  |  |  |
| Dauer des Krankenhausaufenthaltes |  |  |  |  |  |
| Dauer des einliegenden Harnblasenkatheters |  |  |  |  |  |
| Zeit bis zur vollen Genesung |  |  |  |  |  |

9. Bitte beziehen Sie sich auf Ihre gesamte Entscheidung zur Behandlung des Prostatakrebses.

Bitte geben Sie zu jeder Aussage an, welche Antwort am ehesten auf Sie persönlich zutrifft

10. Bitte beziehen Sie sich auf Ihre Entscheidung zur Wahl des operativen Verfahren bei der Therapie des Prostatakrebses.

Bitte geben Sie zu jeder Aussage an, welche Antwort am ehesten auf Sie persönlich zutrifft

**EORTC-PR25**

Patienten klagen oft über die folgenden Symptome und Probleme. Geben Sie bitte an, in welchem

Umfang diese Symptome und Probleme während der letzten Woche bei Ihnen aufgetreten sind.

Markieren Sie bitte die Zahl, die Ihrem Fall am ehesten entspricht.

**Während der letzten Woche...**

**Überhaupt	Wenig	Mäßig	Sehr**

**nicht**

11. Mussten Sie **tagsüber** häufig Wasser lassen?.............................	①	②	③	④

12. Mussten Sie **nachts** häufig Wasser lassen?.................................	①	②	③	④

13. Wenn Sie Harndrang verspürten, mussten Sie sich

dann beeilen, auf die Toilette zu kommen? ………………………..	①	②	③	④

14. Hatten Sie Schwierigkeiten, genug Schlaf zu bekommen, weil

Sie nachts häufig aufstehen mussten, um Wasser zu lassen? ……...	①	②	③	④

15. War es schwierig für Sie, aus dem Haus zu gehen,

weil Sie in der Nähe einer Toilette bleiben mussten? ……………...	①	②	③	④

16. Kam es bei Ihnen zu unwillkürlichem Harnabgang? …………	①	②	③	④

17. Hatten Sie Schmerzen beim Wasserlassen? …………………..	①	②	③	④

18. Beantworten Sie diese Frage bitte nur, wenn Sie eine

Inkontinenzhilfe tragen: Empfanden Sie es als Problem,

eine Inkontinenzhilfe zu tragen? …………………………………..	①	②	③	④

19. Wurden Sie in Ihren täglichen Aktivitäten durch Ihre

Blasenprobleme eingeschränkt? ……………………………………	①	②	③	④

20. Wurden Sie in Ihren täglichen Aktivitäten durch Ihre

Darmprobleme eingeschränkt? …………………………………….	①	②	③	④

21. Kam es bei Ihnen zu unwillkürlichem Abgang von Stuhl? ……	①	②	③	④

22. Hatten Sie Blut im Stuhl? ……………………………………...	①	②	③	④

23. Hatten Sie das Gefühl, dass Ihr Bauch aufgebläht ist? ………..	①	②	③	④

24. Hatten Sie Hitzewallungen? ……………………………………	①	②	③	④

25. Hatten Sie schmerzende oder vergrößerte

Brustdrüsen oder Brustwarzen? ……………………………………	①	②	③	④

16. Hatten Sie Schwellungen in Ihren Beinen oder Knöcheln? ……	①	②	③	④

**Während der letzten 4 Wochen...**

**Überhaupt	Wenig	Mäßig	Sehr**

**nicht**

27. Empfanden Sie die Gewichts**abnahme** als Problem? …………	①	②	③	④

28. Empfanden Sie die Gewichts**zunahme** als Problem? ………….	①	②	③	④

29. Haben Sie sich aufgrund Ihrer Krankheit oder der

Behandlung weniger männlich gefühlt? …………………………...	①	②	③	④

30. Hatten Sie Interesse an Sex? …………………………………...	①	②	③	④

31. Waren Sie sexuell aktiv (mit oder ohne Geschlechtsverkehr)? .. ①	②	③	④

**BEANTWORTEN SIE DIE NÄCHSTEN VIER FRAGEN BITTE NUR, WENN SIE WÄHREND DER LETZTEN 4 WOCHEN SEXUELL AKTIV WAREN**

**Überhaupt	Wenig	Mäßig	Sehr**

**nicht**

32. Konnten Sie Sex genießen? …………………………………….. ①	②	③	④

33. Hatten Sie Schwierigkeiten, eine Erektion zu

bekommen oder aufrechtzuerhalten? ……………………………..... ①	②	③	④

34. Hatten Sie Probleme mit dem Samenerguss

(z.B. trockene Ejakulation)? ……………………………………….	①	②	③	④

35. War Ihnen bei sexueller Intimität oder dem

Gedanken an sexuelle Intimität unwohl? …………………………..	①	②	③	④

36. Wie viele Einlagen oder Inkontinenzvorlagen haben Sie normalerweise in den letzten 4 Wochen am Tag gebraucht, um den Urinverlust unter Kontrolle zu haben?

- Keine
- Eine Einlage am Tag
- Zwei Einlagen am Tag
- Drei oder mehr Einlagen am Tag
